# The importance of multisection sagittal and coronal magnetic resonance imaging evaluation in the assessment of temporomandibular joint disc position

**DOI:** 10.1007/s00784-020-03347-9

**Published:** 2020-06-17

**Authors:** Monika Litko-Rola, Jacek Szkutnik, Ingrid Różyło-Kalinowska

**Affiliations:** 1 Department of Functional Masticatory Disorders, Medical University of Lublin, Karmelicka 7 Street, 20-081 Lublin, Poland; 2 Department of Dental and Maxillofacial Radiology, Medical University of Lublin, Karmelicka 7 Street, 20-081 Lublin, Poland

**Keywords:** Temporomandibular joint disorders, Temporomandibular joint dysfunction syndrome, Temporomandibular joint disc, Magnetic resonance imaging

## Abstract

**Objectives:**

The aim of this study was to evaluate diagnoses of temporomandibular (TMJ) disc displacement by comparing evaluations done on the basis of central sagittal scans only, the most often used in temporomandibular disorder (TMD) patients, with a multisection evaluation done with both sagittal and coronal scans.

**Materials and methods:**

Multisection MRI analysis of 382 TMJs was conducted in 191 patients with disc displacement according to RDC/TMD criteria. Disc position in the intercuspal position (IP) was assessed two times using two different methods. The first method involved a TMJ disc position evaluation on the central slide in the oblique sagittal plane only. In the second method, the TMJ disc position was assessed on all oblique sagittal and coronal images. McNemar’s *χ*^2^ test was used to evaluate the differences between the sensitivities of two methods.

**Results:**

The first method (central oblique sagittal scans assessment) identified 148 TMJs (38.7%) with normal disc position compared with 89 TMJs (23.3%) with normal disc position found by the second method (all oblique sagittal and coronal scans assessment). The sensitivity of analysis in both planes was significantly higher than in the sagittal plane only (*p* < 0.001).

**Conclusions:**

The multisection analysis in the sagittal and coronal plane allows to distinguish the correct disc position from disc displacement and thus improve evaluation of TMJ internal derangement.

**Clinical relevance:**

The multisection sagittal and coronal images should be recommended as a standard in MRI of the TMJ disc displacement in patients with TMD to avoid false-negative diagnoses.

## Introduction

Temporomandibular joint (TMJ) disc displacement is one of the most common types of temporomandibular disorders (TMD) that produce functional disturbances of the masticatory system. Knowledge of TMD has increased along with the further development of diagnostic methods. Currently, magnetic resonance imaging (MRI) of the TMJ is the preferred imaging modality for the diagnosis of disc displacement. MRI is a non-invasive multidimensional technique that produces high-quality images of soft tissues without ionising radiation [1, 2].

Published literature on TMJ disorders most commonly focuses on anterior displacement of the TMJ disc. This may be because in most of these studies, TMJ disc position was analysed only in the sagittal plane [3–9]. Consequently, other directions of disc displacement (e.g. medial, lateral) were not considered. Moreover, according to RDC/TMD image analysis criteria, only sagittal MR images were used as a reference method for the TMJ disc displacement diagnosis in TMD patients despite the fact that standard imaging protocol calls for a series of sagittal and coronal images [10]. Only a few studies focus on disc displacement in the coronal plane [11–13].

Coronal TMJ MRI analysis was introduced in 1986 by Katzberg et al. and is still routinely performed as a part of the TMJ imaging procedure. Yet, there is little agreement concerning its contribution to the routine evaluation of the disc position in TMD patients [14].

From the anatomical point of view, TMJ is a spatial structure. Depending on the direction of displacement in both the sagittal and coronal planes, various types of displacement can be distinguished—anterior, posterior, lateral, medial and combined [15]. Correct recognition of the full status of disc position is important for various surgical and non-surgical procedures involving disc repositioning techniques as well as for the prognosis and assessment of treatment outcomes [16]. In cases with clinical symptoms of TMD in which orthodontic treatment is planned, the status of the disc-condyle complex should also be assessed since this treatment might have an impact on the relationship of the TMJ structures [17–19].

The approach definition of “normal disc position” based only on the assessment in the sagittal plane used by some authors may be insufficient and becomes quite controversial, especially when only one central image is evaluated.

In the available literature, there is a lack of correlation between TMJ disc position in MRI and clinical symptoms [20, 21]. The compatibility of clinical diagnoses of TMJ disc displacement and diagnoses based on the MRI, according to different authors, depending on the type of displacement, ranges from 44 to 90% [5, 6, 9]. It can be suspected that this may be, to some extent, due to the method of assessing the TMJ disc position in MRI. This discrepancy between imaging findings and symptoms undeniably complicates clinical management.

Hence, the lack of substantial agreement in published data on the advisability of using the wider diagnostic protocol of MRI TMJ disc position assessment in TMD patients is of concern here. A comparison of diagnostic information based on the central scan in the sagittal plane only with that obtained with multisection sagittal and coronal imaging in a large sample of participants is essential. Thus, the aim of this study was to evaluate diagnoses of TMJ disc displacement by comparing evaluations done on the basis of central sagittal scans only with multisection evaluation done with both sagittal and coronal scans. It is hypothesised that analysis of coronal scans can add important diagnostic information to that obtained from central sagittal scans only and can do much to eliminate false-negative diagnoses of TMJ displacement.

## Materials and methods

### Study group

In this study, MRIs of 382 TMJs were analysed retrospectively in 191 patients (148 women, 43 men), aged from 14 to 60 years. The patients were referred to The Department of Functional Masticatory Disorders, the Medical University of Lublin, for diagnostic examination and treatment of TMJ problems. All participants presented with a clinical symptom of temporomandibular disorder (TMJ clicking, TMJ locking, pain in the temporomandibular region) and were clinically diagnosed with disc displacement according to the RDC/TMD criteria [22, 23]. Patients with a history of facial trauma, systemic inflammatory arthritis, TMJ tumour or TMJ surgery were excluded from the study.

This study was approved by the Ethics Committee of the Medical University of Lublin, Poland (no. KE-0254/158/2018).

### Magnetic resonance imaging

**Fig. 1:**
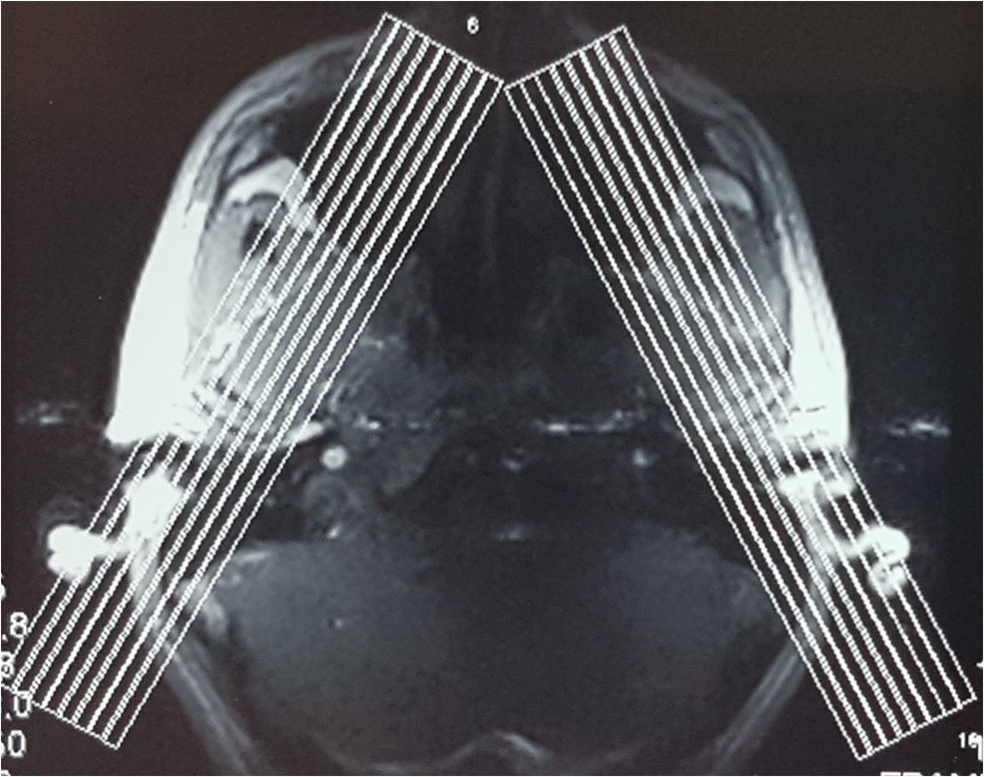
Oblique sagittal slices

**Fig. 2:**
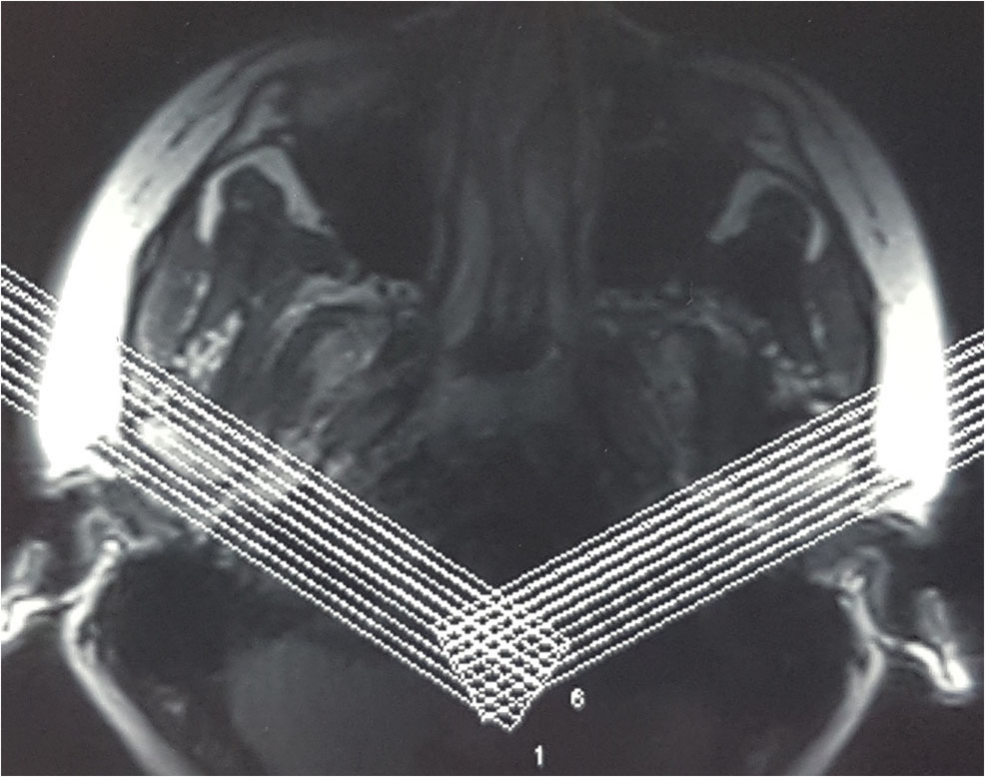
Oblique coronal slices

MRI investigations of the TMJs were carried out in the intercuspal position (IP) using silicon indexes made in this position. All patients underwent bilateral MRI examinations of the TMJ with a TMJ surface coil. MRI was done with the aid of a 1.5-T MRI unit (Eclipse 1.5 T; Picker). PD, T1, T2*-weighted fast spin-echo. MRI was performed in the oblique sagittal and coronal projection. For each TMJ, oblique sagittal images from the medial to the lateral pole and oblique coronal images from the anterior to the posterior pole were obtained (Figs. 1 and 2). The sagittal oblique images were acquired using the following parameters: repetition time = 2000 ms, echo time = 15 ms, field of view 16 cm, slice thickness = 2 mm and matrix size 256 × 256 pixels. For coronal images, the following image acquisition parameters were applied: repetition time = 485 ms, echo time = 12 ms, field of view 14 cm and slice thickness 2 mm.

### Evaluation of TMJ disc position

For disc position evaluation, closed mouth images were analysed to achieve the aim and clarify the results of this study.

MR images were assessed by two experienced observers separately. The observers were evaluated beforehand during a calibration session, at which interobserver reliability assessment revealed acceptable agreement (*κ* = 0.78). In cases of disagreement in the interpretation of the MR images, an assessment was made by consensus.

Disc position in the IP was assessed two times using two different methods. The first method involved a TMJ disc position evaluation on the central image in the oblique sagittal plane only. According to the 12 o’clock criterion, disc position in IP was considered normal if the thickest part of the posterior band was located at the top of the condyle on the 12 o’clock (± 10°). When the posterior band was located anteriorly to this position, the disc was regarded as anteriorly displaced [24].

**Fig. 3:**
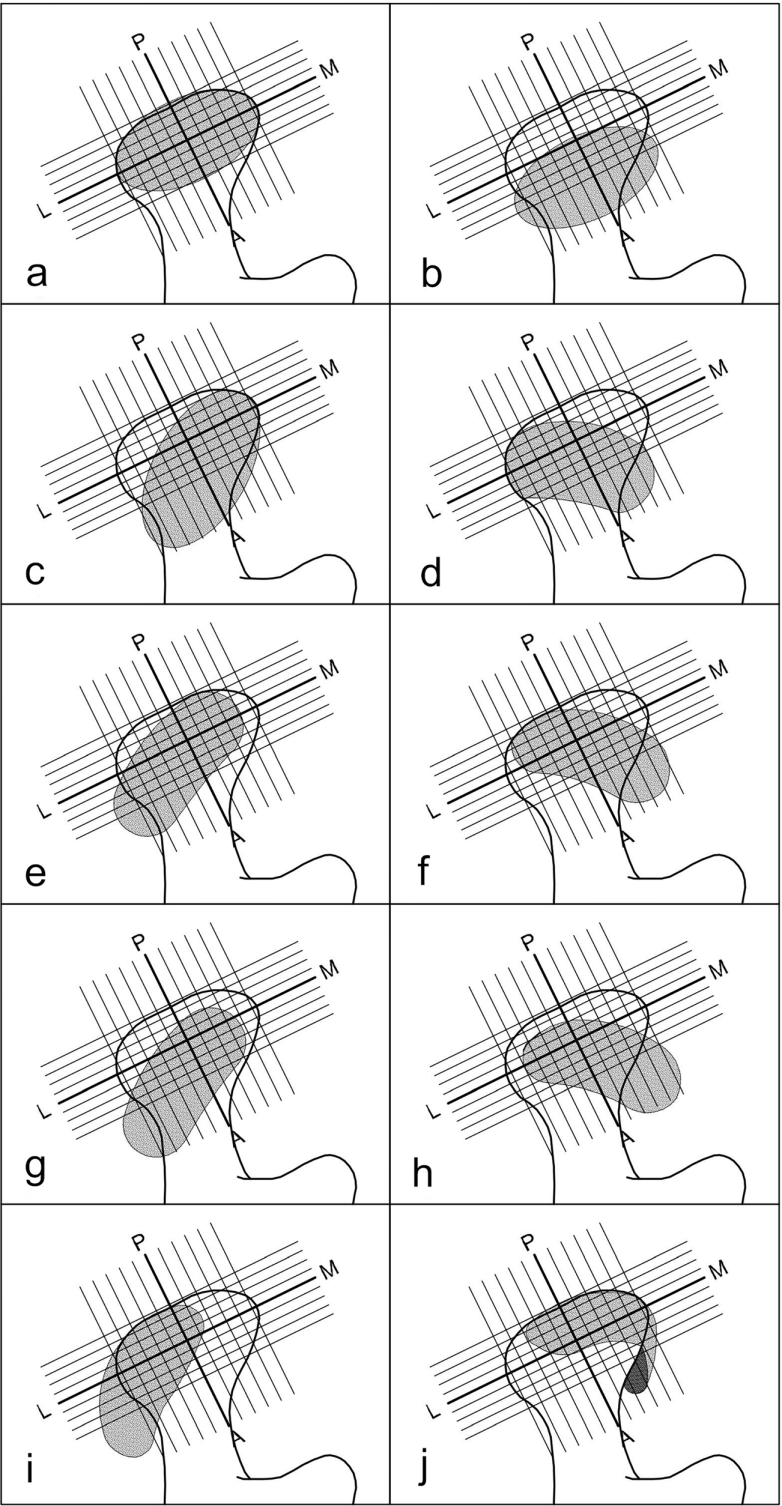
Schemes of TMJ disc position according to multisection sagittal and coronal MRI evaluation: **a** normal superior, **b** complete anterior, **c** partial anterior in the lateral part, **d** partial anterior in the medial part, **e** partial anterolateral, **f** partial anteromedial, **g** complete anterolateral, **h** complete anteromedial, **i** lateral, **j** medial

In the second method, the TMJ disc position was evaluated on all oblique sagittal and coronal images in the IP and categorised according to the literature, as presented in Table 1 and Fig. 3 [24–26].

**Table 1:** TMJ disc position classification according to multisection analysis in sagittal and coronal planes

Disc position	Description
Normal superior	Normal disc position on all oblique sagittal and coronal images
Partial anterior in lateral part	Disc anteriorly displaced on lateral images, otherwise normal
Partial anterior in medial part	Disc anteriorly displaced on medial images, otherwise normal
Complete anterior	Disc anteriorly displaced on all oblique sagittal images, without lateral and medial displacement
Partial anterolateral	Disc anteriorly displaced on lateral images, with lateral displacement
Complete anterolateral	Disc anteriorly displaced on all oblique sagittal images, with lateral displacement
Partial anteromedial	Disc anteriorly displaced on medial images, with medial displacement
Complete anteromedial	Disc anteriorly displaced on all oblique sagittal images, with medial displacement
Lateral	Disc laterally displaced on all oblique coronal images, otherwise normal
Medial	Disc medially displaced on all oblique coronal images, otherwise normal

*TMJ* temporomandibular joint

Disc displacement was defined as lateral or medial when from one-third to one-half of the disc was displaced laterally or medially on the coronal MR slides, respectively [27].

For further analysis, these categories of disc position were aggregated into the four wider subcategories:

- Normal (superior)
- Anterior (anterior displacement without any lateral and medial component)
- Displaced simultaneously in the sagittal and coronal plane—in an anterolateral or anteromedial direction (anterior displacement with lateral or medial component)
- Displaced only in the coronal plane (pure lateral or medial disc displacement)

### Statistical analysis

The interobserver reliability of measurements was assessed using kappa statistic (Cohen’s kappa, *κ*). McNemar’s *χ*^2^ test was used to evaluate the differences between the sensitivities of two methods of disc position diagnosis. Test *z* was applied to assess the differences between the prevalences.

The statistical analysis was performed using SPSS v. 20.0 and the level of significance was set at 0.05.

## Results

The analysis of 382 MR scans of the TMJs taken of 191 patients in the sagittal plane only detected normal superior disc position in 38.7% and anterior disc displacement in 61.3 % (Table 2).

**Table 2:** Distribution of TMJ disc position in maximum intercuspal position in analysis based on a central slice in sagittal plane only according to category (*n* = 382)

TMJ disc position	TMJ right	TMJ left	Total	
	Number (*n*)	Number (*n*)	Number (*n*)	Percentage (%)
Normal superior	73	75	148	38.7
Anterior	118	116	234	61.3
Total	191	191	382	100.0

*TMJ* temporomandibular joint

**Fig. 4:**
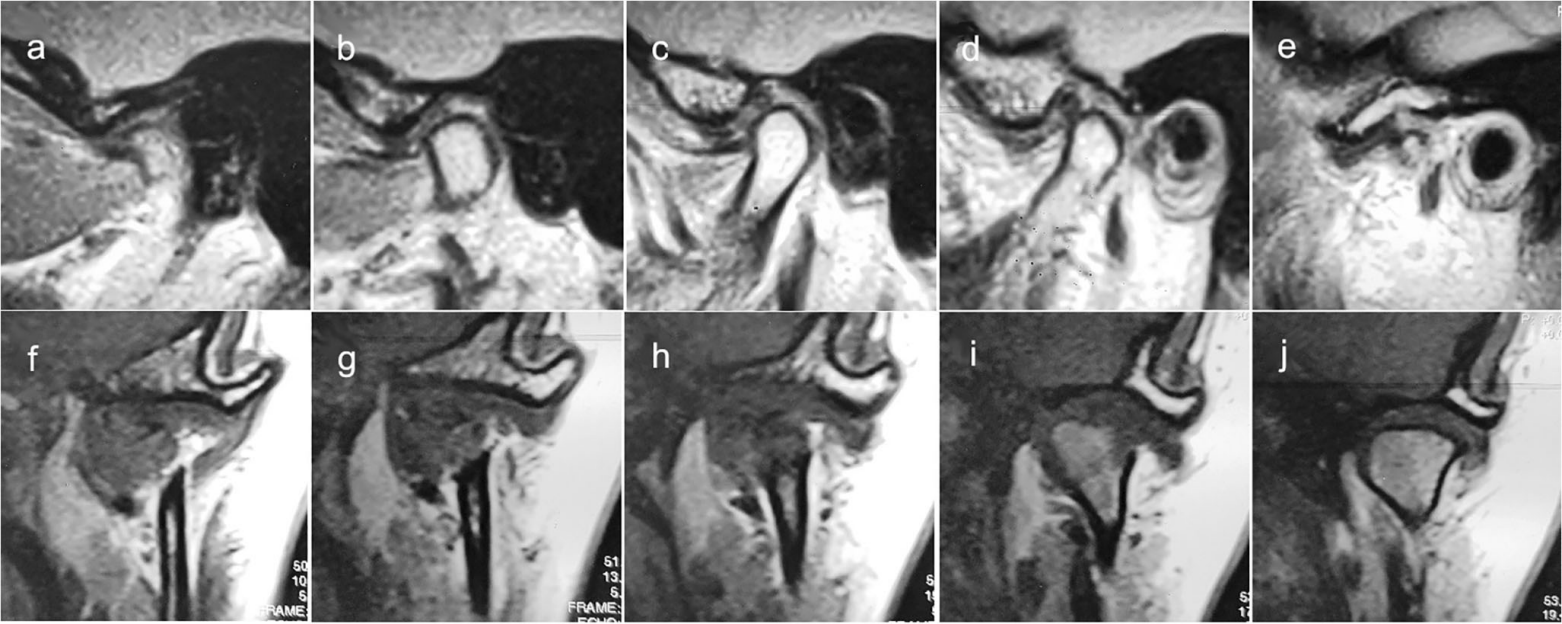
MRI of TMJ with partial anterior disc displacement in the lateral part. **a**–**e Oblique sagittal slices in intercuspal position**. **f**–**j Oblique coronal slices in intercuspal position**

**Fig. 5:**
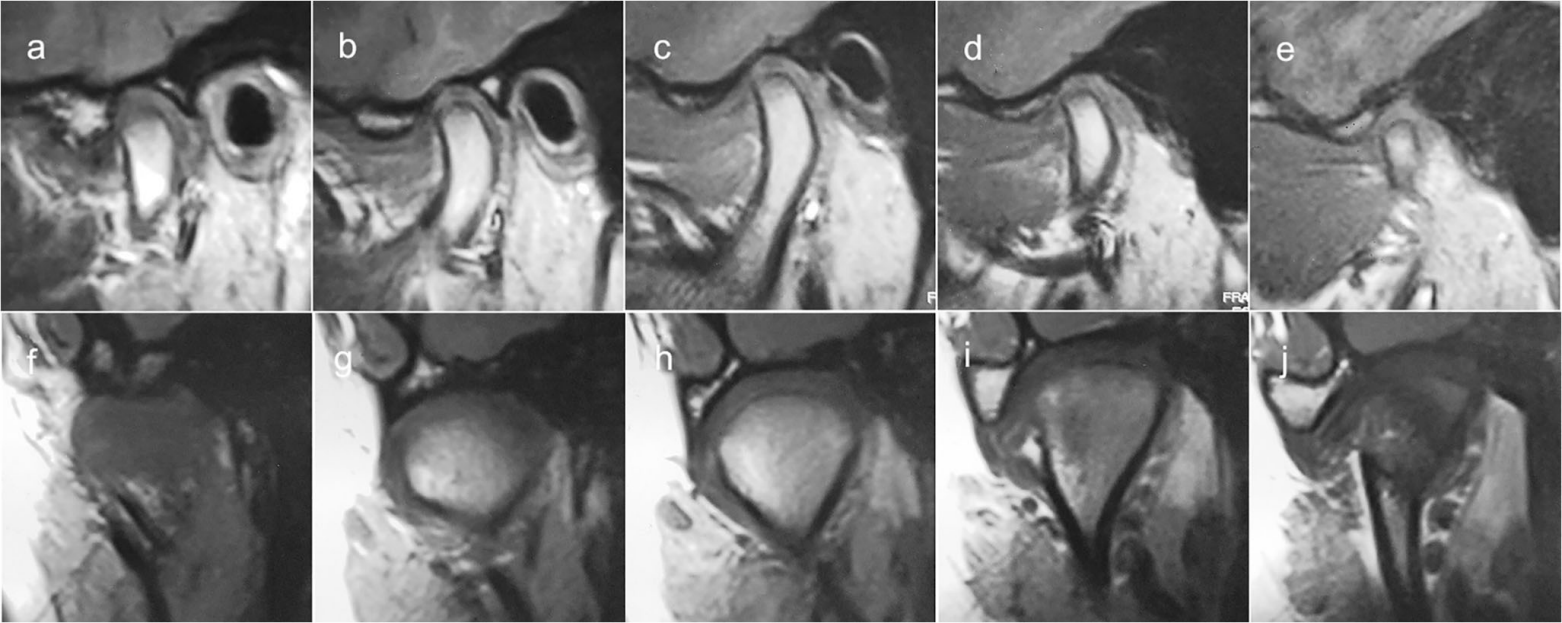
MRI of TMJ with lateral disc displacement. a–e Oblique sagittal slices in intercuspal position. **f**–**j Oblique coronal slices in intercuspal position**

With the use of multisection sagittal and coronal evaluation among the 382 TMJs, normal disc position was detected in 89 (23.3%) cases, partial anterior in the lateral part in 22 (5.8%) cases, partial anterior in the medial part in 3 (0.8%), complete anterior in 74 (19.4%), partial anterolateral in 25 (6.5%), complete anterolateral in 67 (17.5%), partial anteromedial in 7 (1.8%), complete anteromedial in 36 (9.4%), lateral in 17 (4.5%), and medial in 42 (11.0%) (Table 3, Figs. 4 and 5).

**Table 3:** Distribution of TMJ disc position in maximum intercuspal position multisectionally analysed in sagittal and coronal plane according to category (*n* = 382)

TMJ disc position	TMJ right	TMJ left	Total	
	Number (*n*)	Number (*n*)	Number (*n*)	Percentage (%)
Normal superior	39	50	89	23.3
Partial anterior in the lateral part	13	9	22	5.8
Partial anterior in the medial part	0	3	3	0.8
Complete anterior	37	37	74	19.4
Partial anterolateral	11	14	25	6.5
Complete anterolateral	33	34	67	17.5
Partial anteromedial	4	3	7	1.8
Complete anteromedial	20	16	36	9.4
Lateral	11	6	17	4.5
Medial	23	19	42	11.0
Total	191	191	382	100.0

*TMJ* temporomandibular joint

Based on only sagittal images, anterior disc displacement was found in 234 joints (Table 3). The multisection analysis in both planes confirmed this diagnosis in 99 joints and simultaneous disc displacement in the sagittal and coronal plane was found in 135 joints. This difference was statistically significant (*z* = 3.06; *p* < 0.01; Table 4).

**Table 4:** Comparison of distribution of TMJ disc position in maximum intercuspal position analysed by two methods according to subcategory

TMJ disc position	Analysis based on a central slice in sagittal plane only	Multisection analysis in sagittal and coronal plane
	Number (*n*)	Number (*n*)
Normal superior	148	89
Anterior	234	99*
Displaced in the sagittal and coronal plane	0	135*
Displaced only in the coronal plane	0	59
Total	382	382

*TMJ* temporomandibular joint

*z = 3.06; *p* < 0.01

The first method (central oblique sagittal scans assessment) identified 148 TMJs (38.7%) with normal disc position compared with 89 TMJs (23.3%) with normal disc position found by the second method (all oblique sagittal and coronal scans assessment, Table 5).

**Table 5:** Distribution of MRI diagnoses according to different criteria of analysis of TMJ disc position (*n* = 382)

Criterion		Multisection analysis in the sagittal and coronal plane		Total
		Normal disc position	Disc displacement	
Analysis based on a central slice in the sagittal plane only	Normal disc position	89	59	148
	Disc displacement	0	234	234
Total		89	293	382

*TMJ* temporomandibular joint, *MRI* magnetic resonance imaging

McNemar’s *χ*^2^ = 59.0; *p* < 0.001

On the basis of only central sagittal images, 59 joints (16%) with pure displacement in the coronal plane were diagnosed as normal (Table 5). The sensitivity of multisection analysis in both planes was significantly higher than in the sagittal plane only (*p* < 0.001; Table 5).

## Discussion

Previous papers show that anterior disc displacement may occur alone or together with lateral or medial disc displacement [26, 28]. Nonetheless, many studies on MRI of TMJ consider disc position classifications based primarily on assessment in the sagittal plane to be the most clinically crucial [10]. This approach seems unreasonable, however, since clinical emphasis on anterior disc displacement does not consider other types of disc displacement. Moreover, studies by Eberhard et al. and Kleinrok et al. showed that disc displacement in the coronal plane may also be attributed to painful TMD symptoms [11, 13]. Likewise, the authors’ previous study showed that the severity of disc displacement in both sagittal and coronal planes is also a significant predictor of disc reduction ability during mouth opening, something that can be clinically relevant [29]. Other studies show strong evidence that knowledge of TMJ disc status is a significant factor to be considered also in orthodontic treatment planning due to the possible implications of disc displacement in mandibular growth [30, 31]. The results obtained by Almasan et al. suggested that disc displacement could generate changes of the condyle orientation in the coronal plane [32]. Ikeda and Ikeda noticed that predilection for the lateral part of the joint in incipient disc displacement may have etiologic implications in young pre-orthodontic patients [33].

It is important for the maxillofacial radiologist and clinicians to detect even the earliest mild signs of TMJ derangement in MRI. Therefore, the purpose of the current study was firstly to determine the possibilities and limitations of examination of TMJ disc position using MRI in the sagittal plane only and secondly to determine the value of additional analysis in the coronal plane in a large sample of patients with TMD. The results of this study showed limitations and mistakes in the diagnosis of disc position based solely on the selected central slides in the sagittal plane. It also showed that this approach allowed a significant percentage of disc displacements to be unnoticed.

In present studies according to the first methods based only on the central sagittal images, normal superior disc position was found in 148 cases (38.7%). On the other hand, multisection analysis in both planes confirmed normal disc position in only 89 cases (23.3%) showing that 59 cases with pure lateral or medial disc displacement in the coronal plane were overlooked or falsely assessed as normal compared to the first method of assessment. Applying the McNemar’s *χ*^2^ test, the authors detected a statistically significant difference between sensitivities of both methods used to assess the disc position (*p* < 0.001).

Furthermore, among the 234 TMJ discs recognised on the basis of the first method of assessment as anterior displaced, this diagnosis was confirmed in only 99 cases after analysis in both planes. In 135 cases, this diagnosis was not complete because it did not recognise the lateral or medial component of this displacement. Similar observations of Tasaki and Westesson showed that coronal images helped to avoid a false-negative diagnosis in 13% (*n* = 7) of the joints [34]. Thus, the authors conclude that sensitivity of analysis in both planes is significantly higher than only in the sagittal plane. The lack of consideration given to analysing the MRI in the coronal plane could lead to an improper diagnosis of the TMJ internal derangement in terms of disc displacements.

It should be emphasised as the result of the current study that the method of multisection assessment of disc displacement in the sagittal plane plays an important role in the accuracy of the diagnosis. In the present study, disc position was analysed on many MRI slides showing all portions of the joint. Therefore, employing an increased number of the slides might affect the reliability level in detecting the disc position. This is a different approach compared to other studies which applied one or a few representative slides from all images [3, 6, 10, 21, 35, 36]. Some authors evaluated the sagittal image localised only in the centre of the condyle which they believed best depicted the TMJ’s internal structures [7, 10]. However, even though Ahmed, in an analysis of the image criteria for TMD, assessed only the central slide on MRI in the sagittal plane, he suggested that evaluation of multiple slides would have better reflected clinical situations for a total assessment of the TMJ [10].

Attention should be also paid to inconsistencies in the research methodology of studies in which, despite the fact that its methodology states that the MRI was performed in both sagittal and coronal planes, the position of the disc was evaluated in the sagittal plane only without using the full diagnostic capabilities of the MRI [5, 37, 38].

The assessment of single selected or limited slides in some studies can give a false impression of a correct disc position because, as the present study showed, the disc can be displaced anteriorly exclusively in the lateral or medial of the joint. Analysis of many images from the lateral through central to the medial allows for the elimination of false negative diagnoses of normal disc position. Also, in cases with anterior disc displacement in the lateral or medial part, simultaneous lateral or medial displacement can be suspected. Only examination in the coronal plane can confirm or exclude it.

The present study acknowledged also that in a pure medial or lateral disc displacement, sagittal images can be of limited value and these displacements could be identified from coronal views only. In these cases, sagittal images may not be sufficient because there may be no disc tissue present over the head of the condyle. Only coronal images can visualise this type of displacement [34].

Finally, the current study confirmed that the use of sagittal views only will produce a higher number of false-negative and false-positive diagnoses compared to the evaluation where coronal views were also available. Thus, the results show that the combination of many lateral, central (midcondyle) and medial sagittal images and coronal images will provide a dimensional interpretation of the anteromedial, anterolateral or pure medial and lateral disc displacements. The MRI assessment of a central slice in sagittal plane exclusively is of limited value in determining the true position of the TMJ disc.

The present study drew attention to one more question, namely the big discrepancy in research results between studies based on clinical diagnosis of disc displacement and those based on MRI. The disagreement in studies may be due to differences in patients’ sample, but it can also be the consequence of data interpretation. RDC/TMD protocol together with DC/TMD is easy and fast to use but might lead to an inclusion of similar non-disc displacement-related disorders. Their inclusion can be the result of a clinical exam which does not distinguish symptomatic hypermobility from reciprocal click. Correlating different types of clicks during all mandibular movements with only anterior disc displacement on MRI may contribute to these results [6, 9].

In the current study, the diagnosis of normal disc position in 89 joints, which according to the RDC were qualified for the study based on clinical symptoms as TMJ with disc displacement, suggests incorrect clinical diagnosis. However, there are limitations on this study of the subject because of the retrospective nature of these investigations, and because the clinical protocol for TMD could not be controlled.

Nevertheless, it is clear TMJ imaging in combination with clinical examination is important for diagnosing TMD. There is, therefore, a need to clarify and unify criteria for assessing the TMJ position especially in studies comparing the MRI with clinical symptoms of TMJ displacement. Clinical symptoms should be related to TMJ images in both planes to help resolve the complex relationships between clinical and MRI diagnoses of disc displacement. The current low level of heterogeneity in available studies does not provide clinically significant conclusions [2].

The insights of the present research coincide with the observations by Manfredini et al. who investigated the relationship between click sounds and the TMJ disc position in MRI. It was their view that future studies on this issue should take medio-lateral aspects of TMJ disc displacement into consideration in order to increase study comparability [39]. This would improve the diagnostic agreement between clinical assessment and magnetic resonance imaging for particular categories of disc displacements. This approach could eliminate present controversies on the correlation between MRI and particular clinical parameters.

## Conclusions

The results of the present study indicate there is high diagnostic value in a multisection evaluation of TMJ disc position in the sagittal and coronal plane in TMD patients. Multisection analysis will distinguish the correct disc position from disc displacement. Therefore, to improve evaluation of TMJ internal derangement and avoid false-negative diagnoses, sagittal and coronal images should be recommended as a standard in MRI of the TMJ disc displacement in patients with TMD.
